# Chronic Myeloid Leukemia Stem Cell Biology

**DOI:** 10.1007/s11899-012-0121-6

**Published:** 2012-03-31

**Authors:** Leslie A. Crews, Catriona H. M. Jamieson

**Affiliations:** 1 Department of Medicine, Stem Cell Program and Moores Cancer Center, University of California, San Diego, La Jolla, CA 92093 USA; 2 Department of Medicine, Moores Cancer Center, University of California San Diego, 3855 Health Sciences Drive, La Jolla, CA 92093-0820 USA

**Keywords:** Chronic myeloid leukemia, CML, Leukemia stem cell, LSC, BCR-ABL, Hematopoiesis, Hematopoietic progenitors, Blast crisis, Malignant, Hematologic malignancies, Tyrosine kinase inhibitors, TKI, Imatinib, Therapeutic resistance, RNA, Splicing, Microenvironment, Bone marrow niche, Quiescence, Cell cycle, Signal transduction, Shh, BCL2, Survival, Self-renewal

## Abstract

Leukemia progression and relapse is fueled by leukemia stem cells (LSC) that are resistant to current treatments. In the progression of chronic myeloid leukemia (CML), blast crisis progenitors are capable of adopting more primitive but deregulated stem cell features with acquired resistance to targeted therapies. This in turn promotes LSC behavior characterized by aberrant self-renewal, differentiation, and survival capacity. Multiple reports suggest that cell cycle alterations, activation of critical signaling pathways, aberrant microenvironmental cues from the hematopoietic niche, and aberrant epigenetic events and deregulation of RNA processing may facilitate the enhanced survival and malignant transformation of CML progenitors. Here we review the molecular evolution of CML LSC that promotes CML progression and relapse. Recent advances in these areas have identified novel targets that represent important avenues for future therapeutic approaches aimed at selectively eradicating the LSC population while sparing normal hematopoietic progenitors in patients suffering from chronic myeloid malignancies.

## Introduction

Diagnostic and therapeutic strategies that predict and prevent cancer progression represent compelling unmet medical needs. While advanced cancers are diverse in phenotype, they often share essential functional properties ascribed to stem cells such as the capacity to become quiescent, acquire multi-lineage differentiation potential, survive and self-renew [1]. Early insight into the molecular pathogenesis of cancer stemmed from the discovery of activating oncogenes such as the Philadelphia chromosome positive (Ph^+^), and its constitutively active BCR-ABL protein tyrosine kinase product, in chronic myeloid leukemia (CML) [2–4]. CML represents an important paradigm for understanding the molecular evolution of cancer because it was the first cancer shown to be initiated at the hematopoietic stem cell level by BCR-ABL; the first cancer found to undergo blastic transformation following malignant reprogramming of committed progenitors [5, 6]; and the first target of molecular therapy (eg, imatinib, dasatinib) based on targeting pathologically activated kinases such as BCR-ABL [7].

While the current CML prevalence of 24,000 affected patients in the United States (http://www.leukemia-lymphoma.org) is relatively low, it is expected to increase significantly over the next 20 years as a result of widespread use of BCR-ABL tyrosine kinase inhibitor (TKI) therapy. Previous reports showing preclinical and clinical data demonstrate that discontinuation of TKI therapy results in resurgence of disease [8•], suggesting that quiescent BCR-ABL1-expressing progenitors with enhanced self-renewal and survival capacity persist and drive disease progression [9]. Unfortunately, a growing proportion of patients are intolerant or non-compliant either because they become inured to the fact that they have a potentially fatal disease or cannot afford expensive TKI therapy. Ultimately, a significant percentage of patients are expected to develop TKI resistance driven by quiescent leukemia stem cells (LSC). This has provided the impetus for developing sensitive LSC molecular detection systems as well as LSC targeted therapeutic elimination strategies. The development of new clinical and therapeutic approaches aimed at detection and elimination of LSC that fuel therapeutic resistance and blastic transformation will represent an important step forward in treating patients with CML and related disorders.

**Fig. 1:**
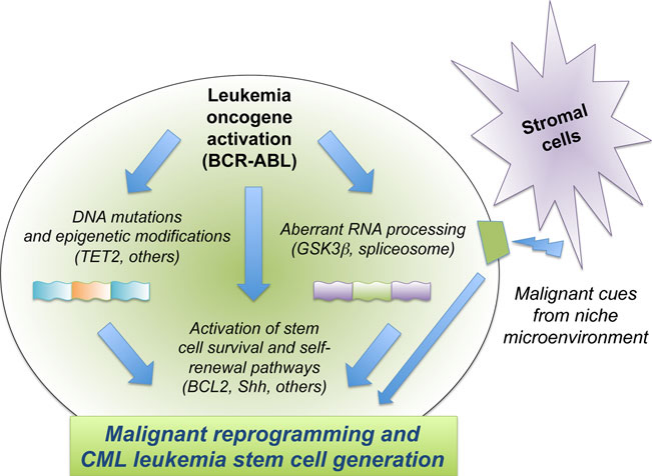
Schematic diagram of molecular mechanisms driving malignant transformation of hematopoietic progenitors into leukemia stem cells in chronic myeloid leukemia

Malignant progenitors play an integral role in disease progression and therapeutic resistance in CML, and the molecular events that are responsible for the generation and maintenance of quiescent and therapeutically recalcitrant, or resistant, LSC have been a topic of intense investigation. Malignant reprogramming of progenitors can occur via abnormal activation of signal transduction pathways and epigenetic events that regulate survival, differentiation, and self-renewal (Fig. 1). Blast crisis (BC) LSC evolve from an expanded myeloid progenitor pool that gains the capacity to self-renew, become dormant and survive in select hematopoietic niches under malignant conditions—characteristics that underlie their particular resilience to therapeutic treatments. In recent years, much effort has been expended both preclinically and in clinical trials toward developing and testing therapeutic approaches aimed at selectively eradicating the LSC population while sparing the normal hematopoietic stem cells (HSC) in patients suffering from chronic myeloid malignancies.

## CML BC LSC Drive Disease Progression and Therapeutic Resistance

Previous studies indicate that BCR-ABL-positive progenitors persist in CML patients despite TKI therapy [10]. This may be attributable in part to quiescent LSC residing in protective niches that acquire additional mutations over time and evade therapeutic treatments. Moreover, bidirectional signaling between LSC and the bone marrow niche might promote malignant cues in LSC-supportive hematopoietic microenvironments characterized by inflammatory signaling and stimulation of cytokine production and interferon pathways that facilitate the reprogramming of progenitors to evolve into BC LSC. Thus, this unique population of dormant progenitors represents an important cellular basis of disease relapse derived from a resurgence of therapeutically recalcitrant LSC, and therapies aimed at eradicating these cells could represent an important step forward toward curbing therapeutic resistance. The molecular mechanisms governing the evolution of normal HSC into malignant progenitors have not been completely elucidated. While BC LSC are known to evolve from an expanded myeloid progenitor pool with enhanced survival and self-renewal abilities and acquired quiescence, these cells are distinct from the chronic phase (CP) CD34^+^CD38^−^ population that has been previously considered as CML LSC. Recent data suggest that deregulation of pathways governing progenitor quiescence, survival and self-renewal, as well as epigenetic alterations in specific hematopoietic niches, fuel BC LSC persistence and therapeutic resistance.

Evidence suggests that patients with advanced CML develop resistance rapidly and generally do not achieve durable complete molecular remission with single-agent TKI therapy as a result of a failure of imatinib to eradicate LSC [10, 11]. This dormant population of cells is relatively resistant to traditional cytotoxic chemotherapies and molecular therapies that target rapidly dividing cells in CML, and thus constitutes an important reservoir of cells that triggers resurgence of disease. Another issue that needs to be addressed is the possibility that adding a new molecular inhibitor to existing treatment with TKI could prove clinically useful. However, determining the precise molecular targets that would be most effective to pursue using combination therapeutic strategies requires a comprehensive understanding of the molecular mechanisms that drive the induction and maintenance of the quiescent and therapeutically resistant LSC population.

## Quiescent BC LSC Persist and Contribute to the Progression of CML

Although the precise molecular mechanisms fueling the generation of treatment-resistant LSC remain elusive, it is quite possible that one critical factor might be deregulation of the cell cycle, which may promote quiescence of LSC that allows this population to evade therapies targeting metabolically active cells. The normal progression of cells through the various stages and checkpoints that guide this process requires the precise regulation of a complex choreography of molecular events involving cyclins, cyclin-dependent kinases, and their negative regulators including p27(kip1) and p21(cip1). Under homeostatic conditions, the activities of these components are tightly regulated. In contrast, abnormal tyrosine kinase-directed phosphorylation of cell cycle regulatory proteins and mis-localization of cell cycle proteins have been implicated in de-regulation of the cell cycle in BCR-ABL expressing cells [12]. Additionally, signal transduction molecules implicated in cell survival and self-renewal—two critical characteristics of quiescent LSC—have been linked to key regulators of the cell cycle.

It is unclear why under certain conditions this may promote uncontrolled proliferation while in other contexts cell quiescence may be favored. However, it is possible that cues from the microenvironment might be involved. Several lines of evidence support the contention that LSC localized to the bone marrow niche are dormant and resistant to traditional cytotoxic therapies. Specific signals from the surrounding stromal cells might promote LSC cell cycle arrest and allow them to persist even during treatment with TKI therapies. Altered interactions between CML LSC and the bone marrow microenvironment can occur as a result of direct intercellular contact; secreted factors such as cytokines or chemokines; or through microenvironmental matrix proteins that regulate cellular adhesion and migration, and growth factor receptor and integrin signals [13], among other activities. Recent evidence provides new insight into the potential contribution of the microenvironment to the initiation and progression of myeloid disorders and leukemia, and may provide a unique area for the development of combination therapeutic strategies aimed at eradicating the resistant LSC population in CML.

## Maintenance of LSC in Supportive Hematopoietic Niches

Previous reports support the hypothesis that LSC fuel therapeutic resistance, relapse, and disease progression from CP to BC as a result of enhanced survival and self-renewal combined with a propensity to become dormant in supportive microenvironments [14]. To date, therapies aimed at eradicating quiescent LSC while sparing normal HSC function have remained elusive [14]. Development of such therapeutic strategies targeting LSC in supportive niches would represent an important complement to current standard of care TKI treatments. Recent studies have made strides toward elucidating the role of the hematopoietic microenvironment in LSC generation and maintenance. Under physiological conditions, normal HSC residing in the bone marrow niche benefit from cell-extrinsic support derived from the heterogeneous cell populations that comprise the surrounding hematopoietic microenvironment. The cell types present in this unique environment include primarily cells of mesenchymal lineages (osteoblasts and other progenitors, osteocytes), endothelial and perivascular cells, and adipocytes [15]. Together with the HSC that reside in the bone marrow, these cell populations exhibit bidirectional signaling that suggests the existence of a functional partnership between HSC and the resident stromal cells. Thus, in the development and progression of CML, defects in either LSC or the surrounding microenvironmental cells could deregulate critical cellular pathways and impair the normal process of hematopoiesis.

The role of the hematopoietic microenvironment in the development and progression of leukemic disorders has been a subject of intense investigation [15, 16•, 17•]. Previous studies suggest that cell-intrinsic defects drive myeloid progenitor expansion and malignant reprogramming in CML, while more recent data favor a primary role for a defective microenvironment in the generation of LSC [16•]. This presents an opportunity for consideration of the relative contributions of “nature versus nurture” to leukemic development at the microenvironmental level.

Seminal mouse transgenic studies reveal that niche-induced oncogenesis can drive the development of myelodysplasia [16•]. Interestingly, genetic manipulation of specific subsets of stromal cells can alter differentiation, proliferation, and apoptosis of heterologous cells. Specifically, deletion of Dicer1 in mouse osteoblast progenitors results in disruption of homeostasis and development of a myelodysplastic phenotype in deficient mice [16•]. Strikingly, transplantation of normal HSC from wild-type mice into the *Dicer1* deleted mice also resulted in the development of myelodysplasia in the recipient animals. This supports a critical role for perturbations in the bone marrow microenvironment in the development of disorders of myeloid progenitors, however disease initiation and progression to BC driven by alterations intrinsic to malignant CML progenitors has also been well characterized.

The truth likely lies somewhere in between the two poles of niche-induced versus LSC-autonomous oncogenesis, wherein subtle changes in both HSC and the bone marrow microenvironmental stromal cells act in concert to drive generation of LSC and persistence of disease, and further exacerbate aberrant regulation of cellular pathways. Malignant CML progenitors harboring mutations and epigenetic alterations that drive enhanced survival and self-renewal might perpetuate pro-leukemic signals from the microenvironment via abnormal inter-cellular signaling with stromal cell populations. Moreover, recent reports indicate that imatinib treatment promotes migration of CML progenitors to the bone marrow via activation of inflammatory signaling receptors (eg, CXCR4), which fosters the survival of quiescent LSC in the bone marrow niche [18].

## Aberrant Regulation of Signal Transduction Drives Malignant Reprogramming of CML Progenitors

The precise molecular mechanisms driving malignant reprogramming of progenitors into LSC in BC CML have remained elusive. Myeloid LSC evolve from granulocyte-macrophage progenitors (GMP) that aberrantly activate self-renewal pathways and drive therapeutic resistance [6, 11, 19, 20]. These critical stem cell signaling pathways represent important alternative targets for development of molecular therapies that might subdue the resistant LSC population and prove to be useful in the clinic in combination with current TKI treatments. Previous studies established the concept of malignant progenitor reprogramming through subversion of stem cell pathways, such as Wnt/β-catenin [20], which has been shown to play a critical role in the survival of LSC [21] with acquired imatinib resistance [22]. Other components of Wnt signaling pathways have also been implicated in the pathogenesis of CML. While sequencing of human LSC revealed very few alterations at the DNA level, there were three SNPs that predicted splicing of the negative Wnt pathway regulator—glycogen synthase kinase 3β (GSK3β). Targeted RNA sequencing revealed a recurrent misspliced, non-functional isoform of GSK3β that predominated in the GMP population, leading to β-catenin activation and enhanced self-renewal [19].

Recent advances have been made toward targeting these signaling molecules with the aim of eradicating LSC or sensitizing them to TKI therapies. Candidate therapies under development include inhibitors of Wnt/β-catenin signaling. New evidence suggests that such therapeutic approaches may hold promise for targeting imatinib-resistant LSC populations, and may represent an important therapeutic strategy in CML and other leukemic disorders. However, current reports of therapeutic Wnt inhibition in models of CML are limited and will require further in vivo studies to elucidate the therapeutic efficacy of these approaches.

### Deregulation of Cell Survival Pathways in CML LSC and Therapeutic Strategies

Another potential therapeutic target that has received recent attention is cell survival gene pathways that are aberrantly activated in CML LSC. For example, deregulation of the anti-apoptotic B-cell lymphoma/leukemia-2 (BCL2) family of genes contributes to LSC apoptosis resistance in the bone marrow microenvironment. Overexpression of BCL2 family genes has been observed in human BC CML and may fuel LSC survival (Goff et al., unpublished results). Human stem cells express myriad pro-death and pro-survival BCL2 family members, each with alternative splice isoforms, rendering the study of human stem cells essential for predicting both the potential efficacy and toxicity of targeted BCL2 inhibition as a therapeutic strategy to eliminate apoptosis resistant LSC. Recent data demonstrate that human LSC express numerous anti-apoptotic BCL2 family splice isoforms. This family of proteins provides a promising candidate for LSC-targeted strategies aimed at selectively eliminating dormant CML LSC. Since multiple BCL2 family members appear to be involved, it is possible that a pan-BCL2 inhibitor will be required to abrogate LSC survival.

We have recently demonstrated that potent inhibitors of BCL2 pro-survival family proteins significantly inhibit LSC survival, at doses that spare normal hematopoietic progenitors in BC CML (Goff et al., unpublished results). Thus, anti-apoptotic *BCL2* gene overexpression plays a key role in therapeutic resistance of quiescent BC CML LSC. Combination therapies targeting activated kinases in concert with anti-apoptotic factors such as the BCL2 family may represent a key strategy toward preventing therapeutic resistance and may be more broadly applicable to LSC in other malignancies.

### Deregulation of Stem Cell Self-Renewal Pathways in CML LSC and Therapeutic Strategies

Alternative important potential targets for therapeutic development in CML are pathways that regulate stem cell self-renewal. For example, one critical stem cell-associated self-renewal gene that has been implicated in the pathogenesis of myeloid and other malignancies is Shh [23]. Shh activation, through mutations and/or abnormal transcriptional regulation and over-expression of downstream effector molecules such as the GLI family (GLI1, GLI2, GLI3) has been linked to the development of varied tumor types, from those affecting brain, skin, and muscle to cancers in other organs including the pancreas, prostate, lungs, and gastrointestinal tract [24]. Shh signaling plays a role in embryonic patterning and fetal development [25], as well as serving critical functions in adult cell populations such as cell cycle regulation in the hematopoietic system [26].

While in hematological malignancies, previous studies have linked Shh activation to cancer stem cell (CSC) generation and disease progression [27], others have shown that Shh signaling modulates the hematopoietic niche during disease progression [28]. In recent studies we demonstrated that treatment of human BC LSC engrafted immunodeficient mice with a selective Shh inhibitor reduced leukemic burden in a niche-dependent manner commensurate with GLI downregulation (Shih et al., unpublished results). Interestingly, Shh has also been implicated in cell cycle regulatory activities such as cell growth (via cyclin D) and proliferation (via both cyclin D and cyclin E) [29], which may be responsible in part for the malignant potential of abnormal activation of this pathway in adult tissues. While precise regulation of hedgehog signaling pathways plays an indispensable role in normal embryonic development, it is becoming clear that malignant reprogramming driven by abnormal activation of such survival and self-renewal pathways in mature tissues may have severe consequences. It is also essential to keep in mind that none of these individual signal transduction pathways acts in a closed system, and as such, active regulation and cross-talk with other signaling pathways play a dynamic role in the regulation of discrete signaling events. Intriguingly, previous reports have suggested that crosstalk between Shh, Wnt, and pathways involving cell cycle regulators might form an important hub in CML progression [30].

## Activation of Malignant Transcriptional Programs is a Feature of Myeloid Disorders

While thus far we have covered several examples of critical pathways that may drive LSC generation and maintenance, including cell cycle, survival, and self-renewal gene families, the reality is that a plethora of abnormally regulated pathways have been implicated in the pathogenesis of CML. This hints at the possibility that broader reprogramming events at the DNA and/or RNA level may be responsible for the downstream molecular events that drive disease progression. Although abnormal activation of signal transduction pathways promoting cell cycle de-regulation and malignant cues derived from LSC-supportive hematopoietic microenvironments may be responsible in part for the development and progression of CML, other post-transcriptional mechanisms may also contribute. Monumental efforts to date have resulted in the identification of DNA mutations and translocations (eg, BCR-ABL) that constitute the initiating oncogenic events in the development of chronic leukemic disorders, however the persistence of LSC and the rise of therapeutic resistance among patients receiving long-term TKI therapies indicates that other mechanisms may fuel leukemic progression and relapse. This has provided the impetus for elucidating the role of genetic and epigenetic alterations fueling cancer progression.

LSC dormancy, survival, and self-renewal, properties that drive both leukemic transformation and therapeutic resistance, occur as a consequence of cell type-specific and microenvironment-specific alterations in both DNA repair and RNA processing. Important clues to the molecular pathways involved in leukemia initiation and progression have been revealed previously by mutational analyses of tumors that described accumulated DNA mutations. In particular, alterations in TET2—a regulator of cytosine methylation—have been detected frequently in myelodysplastic syndromes (MDS) and more recently have been reported to occur as secondary cytogenetic abnormalities in acute and blast phase CML [31]. While a plethora of studies investigating molecular events that fuel human CML progression have focused on acquired DNA mutations, recent data show that aberrant processing of coding and non-coding RNAs represent an important source of proteomic diversity driving therapeutic recalcitrance and relapse [32•]. Recently, novel mutations in components of the RNA splicing apparatus have been described in MDS [33, 34•, 35], and it is possible that abnormalities in the spliceosome may also contribute to aberrant myeloid development in CML. Together these observations suggest that post-transcriptional modifications are pivotal events in leukemogenesis. Thus, these cellular processes represent a source of malignant epigenetic adaptation that may be responsible for broad functional changes in the LSC transcriptome and proteome, and are worthy of further investigation.

### Abnormal RNA Processing Events in CML Progression

As discussed above, previous studies have implicated the generation of novel splice acceptor sites in critical signaling molecules, such as GSK3β, can result in the generation of novel splice isoforms that promote malignant reprogramming of progenitors into LSC that drive blastic transformation of CML [19, 36]. However, the mechanisms governing aberrant splicing have not been elucidated, and may provide another group of alternative molecular targets for therapeutic development in CML. Notably, alternative splicing of other CSC markers, such as CD44, has been associated with invasive breast cancer while CD47 splice isoforms are overexpressed by CSC in a number of tumors and prevent macrophage-mediated phagocytosis [37].

Alternative splicing also plays a central role in the maintenance of fetal and adult HSC and may have important functions in LSC and disease progression. Cancer-associated alterations in splicing have been shown to affect genes controlling apoptosis (BCL2, *GSK3β*, *Bcl*-*x*, *p53*, and *caspase*-*2*) and drug resistance [38]. One example is the switch in BCL2 family member isoform expression from the pro-apoptotic to pro-survival isoforms that contributes to apoptosis resistance of the LSC and has been described and both observed in both MDS and CML disease progression. As discussed in more detail above, recent RNA sequencing and qRT-PCR data demonstrate that a number of anti-apoptotic BCL2 splice isoforms are highly expressed by human CML LSC (Goff et al., unpublished results). The BCL2 family represents an important therapeutic target and determination of the BCL2 family member isoform expression may be a valuable diagnostic tool for patients with CML and other myeloproliferative neoplasms in the clinic.

Moreover, in CML novel splicing events have been identified in transcripts of other genes involved in the regulation of hematopoietic development and cell fate determination (Ikaros) [39], in cell growth and senescence factors (Gfi1b, hTERT) [40, 41], and even in BCR-ABL itself, an event which may contribute to imatinib resistance [42]. Although somatic cis-acting mutations that affect splicing may contribute to alternative transcript generation in cancer, aberrant expression of splicing factors is one possible source of human neoplasia-associated transcriptome diversity. Notably, recent landmark studies have identified mutations in components of the cellular splicing machinery that are associated with the development of human MDS [33, 34•, 35]. However, it remains to be elucidated whether similar mechanisms might contribute to abnormal splicing activities in CML.

Previous reports have proposed that general changes in pre-mRNA splicing as a result of p210BCR/ABL kinase activity may contribute to CML pathogenesis [43]; however the mechanisms promoting abnormal RNA processing in CML remain to be elucidated. Recent studies reveal that genes with predicted RNA editing events are significantly enriched in cancer-related pathways in breast cancer and glioblastoma, supporting a role for abnormal RNA processing in gene product diversity in cancer pathogenesis [44]. Although increased expression of RNA editing enzymes has been implicated in the mutational evolution of other cancers [45], little is known about the role of RNA editing in human LSC survival, cell fate determination, and self-renewal. Taken together, aberrant RNA processing and splicing may act as critical regulators of LSC generation and maintenance, and perhaps more importantly, it sets the stage for CSC elimination in other human malignancies.

## Conclusions

In the pathogenesis of CML, therapeutically recalcitrant LSC are thought to fuel disease progression and relapse. While targeted TKI therapies (BCR-ABL antagonists) have resulted in improved survival of patients with CP CML, the prevalence of CML doubled in the U.S. between 2001 and 2009 (http://seer.cancer.gov/). Unfortunately, a growing proportion of patients are non-compliant because they become inured to the fact that they have leukemia or simply cannot afford full dose TKI therapy as a result of spiraling annual costs and thus, progress to advanced phase disease with a 5-year survival rate of less than 30 %. Thus, elimination of LSC contributing to therapeutic resistance, the primary cause of cancer death, is of high clinical importance, and understanding the molecular mechanisms of CML LSC biology that drive the generation of LSC and their persistence is crucial.

Characteristic features of CML LSC, such as their enhanced capacity for survival, self-renewal, and persistence over time through dormancy, underlie their unique resilience to conventional therapies. Cell cycle alterations, abnormal activation of signaling pathways involved in cell survival and self-renewal, and microenvironmental cues from the malignant bone marrow microenvironment can facilitate the enhanced survival and malignant transformation of CML LSC. More recently, epigenetic alterations at the RNA level, including aberrant splicing and RNA processing, have been implicated in the pathogenesis of CML and MDS. Therapeutic strategies targeting regulators of cell survival and self-renewal, or RNA processing pathways, could represent vital novel components of a potentially curative strategy for advanced CML that may obviate the need for costly continuous TKI therapy by increasing sensitivity to therapy. Such therapeutic strategies could provide a valuable companion diagnostic with relevance to a broad group of patients suffering from leukemia and lymphoma disorders.

## References

[1] Jamieson C (2009). The MLLgnant consequences of reverting to an embryonic transcriptional program. Cell Stem Cell.

[2] Nowell PC, Hungerford DA (1960). Chromosome studies on normal and leukemic human leukocytes. J Natl Cancer Inst.

[3] Ben-Neriah Y, Daley GQ, Mes-Masson AM (1986). The chronic myelogenous leukemia-specific P210 protein is the product of the bcr/abl hybrid gene. Science.

[4] Shtivelman E, Lifshitz B, Gale RP, Canaani E (1985). Fused transcript of abl and bcr genes in chronic myelogenous leukaemia. Nature.

[5] Fialkow PJ, Jacobson RJ, Papayannopoulou T (1977). Chronic myelocytic leukemia: clonal origin in a stem cell common to the granulocyte, erythrocyte, platelet and monocyte/macrophage. Am J Med.

[6] Jamieson CH, Ailles LE, Dylla SJ (2004). Granulocyte-macrophage progenitors as candidate leukemic stem cells in blast-crisis CML. N Engl J Med.

[7] O’Brien SG, Guilhot F, Larson RA (2003). Imatinib compared with interferon and low-dose cytarabine for newly diagnosed chronic-phase chronic myeloid leukemia. N Engl J Med.

[8] Mahon FX, Rea D, Guilhot J (2010). Discontinuation of imatinib in patients with chronic myeloid leukaemia who have maintained complete molecular remission for at least 2 years: the prospective, multicentre stop imatinib (STIM) trial. Lancet Oncol.

[9] Copland M, Hamilton A, Elrick LJ (2006). Dasatinib (BMS-354825) targets an earlier progenitor population than imatinib in primary CML but does not eliminate the quiescent fraction. Blood.

[10] Graham SM, Jorgensen HG, Allan E (2002). Primitive, quiescent, Philadelphia-positive stem cells from patients with chronic myeloid leukemia are insensitive to STI571 in vitro. Blood.

[11] Minami Y, Stuart SA, Ikawa T (2008). BCR-ABL-transformed GMP as myeloid leukemic stem cells. Proc Natl Acad Sci U S A.

[12] Grimmler M, Wang Y, Mund T (2007). Cdk-inhibitory activity and stability of p27Kip1 are directly regulated by oncogenic tyrosine kinases. Cell.

[13] Chiodoni C, Colombo MP, Sangaletti S (2010). Matricellular proteins: from homeostasis to inflammation, cancer, and metastasis. Cancer Metastasis Rev.

[14] Essers MA, Trumpp A (2010). Targeting leukemic stem cells by breaking their dormancy. Mol Oncol.

[15] Lane SW, Scadden DT, Gilliland DG (2009). The leukemic stem cell niche: current concepts and therapeutic opportunities. Blood.

[16] Raaijmakers MH, Mukherjee S, Guo S (2010). Bone progenitor dysfunction induces myelodysplasia and secondary leukaemia. Nature.

[17] Mendez-Ferrer S, Michurina TV, Ferraro F (2010). Mesenchymal and haematopoietic stem cells form a unique bone marrow niche. Nature.

[18] Jin L, Tabe Y, Konoplev S (2008). CXCR4 up-regulation by imatinib induces chronic myelogenous leukemia (CML) cell migration to bone marrow stroma and promotes survival of quiescent CML cells. Mol Cancer Ther.

[19] Abrahamsson AE, Geron I, Gotlib J (2009). Glycogen synthase kinase 3beta missplicing contributes to leukemia stem cell generation. Proc Natl Acad Sci U S A.

[20] Trowbridge JJ, Moon RT, Bhatia M (2006). Hematopoietic stem cell biology: too much of a Wnt thing. Nat Immunol.

[21] Zhao C, Blum J, Chen A (2007). Loss of beta-catenin impairs the renewal of normal and CML stem cells in vivo. Cancer Cell.

[22] Hu Y, Chen Y, Douglas L, Li S (2009). Beta-catenin is essential for survival of leukemic stem cells insensitive to kinase inhibition in mice with BCR-ABL-induced chronic myeloid leukemia. Leukemia.

[23] Taipale J, Beachy PA (2001). The hedgehog and Wnt signalling pathways in cancer. Nature.

[24] Ruiz i Altaba A, Sanchez P, Dahmane N (2002). Gli and hedgehog in cancer: tumours, embryos and stem cells. Nat Rev Cancer.

[25] Ingham PW (1998). Transducing hedgehog: the story so far. EMBO J.

[26] Trowbridge JJ, Scott MP, Bhatia M (2006). Hedgehog modulates cell cycle regulators in stem cells to control hematopoietic regeneration. Proc Natl Acad Sci U S A.

[27] Radich JP, Dai H, Mao M (2006). Gene expression changes associated with progression and response in chronic myeloid leukemia. Proc Natl Acad Sci U S A.

[28] Olive KP, Jacobetz MA, Davidson CJ (2009). Inhibition of hedgehog signaling enhances delivery of chemotherapy in a mouse model of pancreatic cancer. Science.

[29] Duman-Scheel M, Weng L, Xin S, Du W (2002). Hedgehog regulates cell growth and proliferation by inducing cyclin D and cyclin E. Nature.

[30] Sengupta A, Banerjee D, Chandra S (2007). Deregulation and cross talk among sonic hedgehog, Wnt, Hox and notch signaling in chronic myeloid leukemia progression. Leukemia.

[31] Makishima H, Jankowska AM, McDevitt MA (2011). CBL, CBLB, TET2, ASXL1, and IDH1/2 mutations and additional chromosomal aberrations constitute molecular events in chronic myelogenous leukemia. Blood.

[32] Perrotti D, Neviani P (2007). From mRNA metabolism to cancer therapy: chronic myelogenous leukemia shows the way. Clin Cancer Res.

[33] Yoshida K, Sanada M, Shiraishi Y (2011). Frequent pathway mutations of splicing machinery in myelodysplasia. Nature.

[34] Papaemmanuil E, Cazzola M, Boultwood J (2011). Somatic SF3B1 mutation in myelodysplasia with ring sideroblasts. N Engl J Med.

[35] Hahn CN, Scott HS (2011). Spliceosome mutations in hematopoietic malignancies. Nat Genet.

[36] Perrotti D, Jamieson C, Goldman J, Skorski T (2010). Chronic myeloid leukemia: mechanisms of blastic transformation. J Clin Invest.

[37] Jaiswal S, Jamieson CH, Pang WW (2009). CD47 is upregulated on circulating hematopoietic stem cells and leukemia cells to avoid phagocytosis. Cell.

[38] Rice KN, Jamieson CH (2010). Molecular pathways to CML stem cells. Int J Hematol.

[39] Olivero S, Maroc C, Beillard E (2000). Detection of different ikaros isoforms in human leukaemias using real-time quantitative polymerase chain reaction. Br J Haematol.

[40] Vassen L, Khandanpour C, Ebeling P (2009). Growth factor independent 1b (Gfi1b) and a new splice variant of Gfi1b are highly expressed in patients with acute and chronic leukemia. Int J Hematol.

[41] Drummond MW, Hoare SF, Monaghan A (2005). Dysregulated expression of the major telomerase components in leukaemic stem cells. Leukemia.

[42] Ma W, Giles F, Zhang X (2011). Three novel alternative splicing mutations in BCR-ABL1 detected in CML patients with resistance to kinase inhibitors. Int J Lab Hematol.

[43] Salesse S, Dylla SJ, Verfaillie CM (2004). p210BCR/ABL-induced alteration of pre-mRNA splicing in primary human CD34+ hematopoietic progenitor cells. Leukemia.

[44] Bahn JH, Lee JH, Li G, et al. Accurate identification of A-to-I RNA editing in human by transcriptome sequencing. Genome Res. 2011. 10.1101/gr.124107.111 PMC3246201 21960545

[45] Shah SP, Morin RD, Khattra J (2009). Mutational evolution in a lobular breast tumour profiled at single nucleotide resolution. Nature.

